# Supplementary material for: Albedo changes caused by future urbanization contribute to global warming
Source: Nat Commun. 2022 Jul 1;13:3800. doi: 10.1038/s41467-022-31558-z (PMC9249918; doi:10.1038/s41467-022-31558-z)
Supplement: Supplementary file 1 — Supplementary Information [file 41467_2022_31558_MOESM1_ESM.pdf]

Supplementary Information for

"Albedo Changes Caused by Future Urbanization Contribute to Global Warming"

Zutao Ouyang[1,2*], Pietro Sciusco[2], Tong Jiao[3], Sarah Feron[1,4,5], Cheyenne Lei[2], Fei Li[6], Ranjeet John[7], Peilei Fan[8], Xia Li[9], Christopher A. Williams[3], Guangzhao Chen[10], Chenghao Wang[1], Jiquan Chen[2*]

1. Department of Earth System Science, Stanford University, Stanford, CA, 94305, USA

2. Center for Global Change and Earth Observations and Department of Geography, Environment & Spatial Sciences, Michigan State University, East Lansing, MI, 48823, USA

3. Graduate School of Geography, Clark University, Worcester, MA, 01610, USA

4. Campus Fryslan, University of Groningen, Wirdumerdijk 34, 8911 CE, Leeuwarden, The Netherlands

5. Department of Physics, University of Santiago, Ave Bernardo OHiggins 3363 Santiago de Chile, Chile

6. Grassland Research Institute, Chinese Academy of Agricultural Sciences, Hohhot, 010010, China

7. Department of Biology, and Department of Sustainability, University of South Dakota, Vermillion, SD, 57069, USA

8. Center for Global Change and Earth Observations and School of Planning, Design, and Construction, Michigan State University, East Lansing, MI, 48823, USA

9. Key Lab of Geographic Information Science, School of Geographic Sciences, East China Normal University, Shanghai, 200241, China

10. Division of Landscape Architecture, Faculty of Architecture, The University of Hong Kong, Hongkong, 999077, China

Corresponding authors: Zutao Ouyang, Jiquan Chen.

Email: ouyangzt@stanford.edu, jqchen@msu.edu

Contents

Supplementary Note 1: Seasonal changes of albedo…………………………………………...3
Supplementary Note 2: Diffuse and direct (beam) radiation flux for sensitivity analysis………..4
Supplementary Note 3: Future Snow Cover and radiation……………………………………..5
Supplementary Note 4: Log-normal distribution approximation for equilibrium climate sensitivity (ECS) and radiative forcing (RF)…………………………………………………...6
Supplementary Table 1…………………………………………………………………………7
Supplementary Table 2…………………………………………………………………………9
Supplementary Table 3………………………………………………………………………..10
Supplementary Table 4………………………………………………………………………..11
Supplementary Table 5………………………………………………………………………..12
Supplementary Figure 1……………………………………………………………………….13
Supplementary Figure 2……………………………………………………………………….14
Supplementary Figure 3……………………………………………………………………….15
Supplementary Figure 4……………………………………………………………………….16
Supplementary Figure 5……………………………………………………………………….17
Supplementary Figure 6……………………………………………………………………….18
Supplementary Figure 7……………………………………………………………………….19
Supplementary Figure 8……………………………………………………………………….20
Supplementary Figure 9……………………………………………………………………….21
Supplementary References…………………………………………………………………….22

Supplementary Note 1: Seasonal changes of albedo

To study the albedo-change induced radiative forcing caused by land cover changes is essential to consider the whole annual cycle of albedo as there are substantial changes of land cover especially for vegetative land covers, such as cropland, grassland, and forests (Supplementary Figure 8). There are two major causing factors of seasonal change of albedo: 1) the phenological changes of plants (i.e., the change of greenness, biomass, and canopy) and 2) the change of weather/climate (e.g., radiation and snow).

Particularly, snow cover can quickly make influence as it directly alters the surface conditions. The effect of snow is coupled with urbanization rather than isolated, as when other land covers are converted to urban land, it is changed under both snow-cover and snow-free conditions. It is therefore essential to include the effect of snow cover when computing climate effect induced by annual albedo changes. We thus considered the projections of snow cover under different future climate change scenarios (RCP2.6, RCP4.5, and RCP8.5) in estimating radiative forcing for the future as an essential part of this study.

For such reasons, local scale studies that use summer albedo can potentially produce opposite results of cooling effect from urbanization. For example, Guo et al.[1] reported negative radiative forcing from urbanization in 11 Chinese cities, which is likely because they only used summertime albedo derived from a few Landsat observations. Taking the conversion of cropland to urban land as an example, while cropland albedo is higher than cropland during winter times, locally in summertime it can be lower than urban land at some time windows (Supplementary Figure 9).

Supplementary Note 2: Diffuse and direct (beam) radiation flux for sensitivity analysis

We did not consider future changes in diffuse and direct radiation in our estimate of radiative forcing (RF) mainly because few CMIP5 models produce diffuse and direct radiation outputs. Instead, we analyzed the sensitivity of the estimated RF through the following two experiments.

In the first experiment, we artificially reversed the ratios of diffuse and direct radiation based on contemporary observations from NCEP and used these proportions for future periods. This is an extreme condition as it results in diffuse radiation greater than direct radiation, which is a very unlikely case in most locations. Compared to the original estimates where the ratios of diffuse and direct radiation are assumed constant from current to the future, the relative changes of RF in this experiment are less than 9% in all illustrative scenarios (Supplementary Table 3).

In the second experiment, we focused on possible future changes of diffuse and direct radiation terms based on available CMIP5 projections. Among the 13 CMIP5 models in Appendix B, only three models provide total surface-level downwelling shortwave radiation and diffuse radiation fluxes: CANESM2, NOESM_M, NORESM_ME. We derived the direct beam radiation at the surface using these two flux terms and the solar zenith angle. Note that for solar zenith angle that varies with location and time, we used insolation-weighted zenith angles for all grid points[2] which have been shown to have relatively lower albedo bias than simple average or daytime-weighted zenith angles[3]. The three-model decadal averages (2045-2054 for 2050, and 2090-2100 for 2100) for monthly mean direct and diffuse radiation were then computed for estimating blue-sky albedo and then RF. Compared to the original estimates where the ratios of diffuse and direct radiation are assumed constant from current to the future, the relative changes of RF in this experiment are less than 4% in all illustrative scenarios (Supplementary Table 3).

Supplementary Note 3: Future Snow Cover and radiation

Data for snow cover, surface radiation, and top of atmosphere radiation are based on 13 global climate models (GCMs) from the Coupled Model Intercomparison Project 5 (CMIP5) (see Supplementary Table 4 for a detailed list of selected models). The data were retrieved from the Earth System Grid Federation (ESGF; https://esgf-node.llnl.gov/projects/esgf-llnl/) for three climate scenarios: RCP2.6, RCP4.5, and RCP8.5.

To compute the multi model mean (MMM) of the GCMs, the datasets were resampled to a common 1°×1° spatial grids. Next, for each of the three scenarios, the data were divided into decadal time periods (e.g., 2006-2015 for the reference dataset, and 2025-2034, 2035-2044, etc. for future projections to match urban expansion projects in 2030, 2040, etc.). Monthly climatological snow cover, surface radiation, and top of atmosphere radiation were computed by averaging the monthly values for each decade and scenario, respectively. Finally, the MMM and the multi model standard deviation (SD) was computed for each month and each scenario. Moreover, we bias corrected future snow cover projections using the difference (systematic bias) between predictions and the observed MODIS snow cover during the reference period (i.e., 2006-2015).

Supplementary Note 4: Log-normal distribution approximation for equilibrium climate sensitivity (ECS) and radiative forcing (RF)

Because we only know the mean and the 90% confidence interval for both the adopted ECS and the future RF, we created log-normal distributions to approximate their mean and 90% intervals through numerical simulations. The mean value of the real distribution can be accurately reproduced in the log-normal distributions, but the lower 5% and upper 95% quantiles are both allowed to have ~5-10% differences because there is no exact solution. We finally adopted log normal distributions in Supplementary Table 5 for these quantities used for estimating uncertainties in surface temperature changes.

Supplementary Tables and Figures

Supplementary Table 1 Summary of shared socioeconomic pathway narratives (reprinted from Riahi et al.[4])

SSP1 Sustainability – Taking the Green Road (Low challenges to mitigation and adaptation)
The world shifts gradually, but pervasively, toward a more sustainable path, emphasizing more inclusive development that respects perceived environmental boundaries. Management of the global commons slowly improves, educational and health investments accelerate the demographic transition, and the emphasis on economic growth shifts toward a broader emphasis on human well-being. Driven by an increasing commitment to achieving development goals, inequality is reduced both across and within countries. Consumption is oriented toward low material growth and lower resource and energy intensity.

SSP2 Middle of the Road (Medium challenges to mitigation and adaptation)
The world follows a path in which social, economic, and technological trends do not shift markedly from historical patterns. Development and income growth proceed unevenly, with some countries making relatively good progress while others fall short of expectations. Global and national institutions work toward but make slow progress in achieving sustainable development goals. Environmental systems experience degradation, although there are some improvements and, overall, the intensity of resource and energy use declines. Global population growth is moderate and levels off in the second half of the century. Income inequality persists or improves only slowly and challenges to reducing vulnerability to societal and environmental changes remain.

SSP3 Regional Rivalry – A Rocky Road (High challenges to mitigation and adaptation)
A resurgent nationalism heightens concerns about competitiveness and security, and regional conflicts push countries to increasingly focus on domestic or, at most, regional issues. Policies shift over time to become increasingly oriented toward national and regional security issues. Countries focus on achieving energy and food security goals within their own regions at the expense of broader-based development. Investments in education and technological development decline. Economic development is slow, consumption is material-intensive, and inequalities persist or worsen over time. Population growth is low in industrialized and high in developing countries. A low international priority for addressing environmental concerns leads to strong environmental degradation in some regions.

SSP4 Inequality – A Road Divided (Low challenges to mitigation, high challenges to adaptation)
Highly unequal investments in human capital, combined with increasing disparities in economic opportunity and political power, lead to increasing inequalities and stratification both across and within countries. Over time, a gap widens between an internationally connected society that contributes to knowledge- and capital-intensive sectors of the global economy and a fragmented collection of lower-income, poorly educated societies that work in a labor intensive, low-tech economy. Social cohesion

degrades, and conflict and unrest become increasingly common. Technology development is high in the high-tech economy and industry sectors. The globally connected energy sector diversifies, with investments in both carbon-intensive fuels like coal and unconventional oil, but also low-carbon energy sources. Environmental policies focus on local issues around middle- and high-income areas.

SSP5 Fossil-fueled Development – Taking the Highway (High challenges to mitigation, low challenges to adaptation)

This world places increasing faith in competitive markets, innovation, and participatory societies to produce rapid technological progress and development of human capital as the path to sustainable development. Global markets are increasingly integrated. There are also strong investments in health, education, and institutions to enhance human and social capital. At the same time, the push for economic and social development is coupled with the exploitation of abundant fossil fuel resources and the adoption of resource and energy intensive lifestyles around the world. All these factors lead to rapid growth of the global economy, while global population peaks and declines in the 21st century. Local environmental problems like air pollution are successfully managed. There is faith in the ability to effectively manage social and ecological systems, including by geo-engineering if necessary.

Supplementary Table 2 MODIS IGBP class description (reprinted from Table 3 of MCD12Q1 User's Guide[5])

Value	Class Name	Definition
**1**	Evergreen Needleleaf Forests	Dominated by evergreen conifer trees (canopy >2m). Tree cover >60%.
**2**	Evergreen Broadleaf Forests	Dominated by evergreen broadleaf and palmate trees (canopy >2m). Tree cover >60%.
**3**	Deciduous Needleleaf Forests	Dominated by deciduous needleleaf (larch) trees (canopy >2m). Tree cover >60%.
**4**	Deciduous Broadleaf Forests	Dominated by deciduous broadleaf trees (canopy >2m). Tree cover >60%.
**5**	Mixed Forests	Dominated by neither deciduous nor evergreen (40-60% of each) tree type (canopy >2m). Tree cover >60%.
**6**	Closed Shrublands	Dominated by woody perennials (1-2m height) >60% cover.
**7**	Open Shrublands	Dominated by woody perennials (1-2m height) 10-60% cover.
**8**	Woody Savannas	Tree cover 30-60% (canopy >2m).
**9**	Savannas	Tree cover 10-30% (canopy >2m).
**10**	Grasslands	Dominated by herbaceous annuals (<2m).
**11**	Permanent Wetlands	Permanently inundated lands with 30-60% water cover and >10% vegetated cover.
**12**	Croplands	At least 60% of area is cultivated cropland.
**13**	Urban and Built-up Lands	At least 30% impervious surface area including building materials, asphalt and vehicles.
**14**	Cropland/Natural Vegetation Mosaics	Mosaics of small-scale cultivation 40-60% with natural tree, shrub, or herbaceous vegetation.
**15**	Permanent Snow and Ice	At least 60% of area is covered by snow and ice for at least 10 months of the year.
**16**	Barren	At least 60% of area is non-vegetated barren (sand, rock, soil) areas with less than 10% vegetation.
**17**	Water Bodies	At least 60% of area is covered by permanent water bodies.

Supplementary Table 3 Sensitivity of radiative forcing (RF) to varying diffuse and direct radiation

Scenarios	Periods	RF	RF-Reverse	Change (%)	RF-CMIP5	Change (%)
SSP1-2.6	2050-2018	0.001281	0.001174	-8.40958	0.001327	3.540117
SSP1-2.6	2100-2018	0.001570	0.001434	-8.65681	0.001628	3.714026
SSP2-4.5	2050-2018	0.001326	0.001217	-8.21276	0.001373	3.592404
SSP2-4.5	2100-2018	0.001866	0.001718	-7.90981	0.001929	3.405216
SSP5-8.5	2050-2018	0.001575	0.001449	-8.04681	0.001629	3.425881
SSP5-8.5	2100-2018	0.003068	0.002861	-6.74607	0.003167	3.220061

RF: the RF estimates based on NCEP diffuse and direct radiation ratios both for the present (2018) and for the future (2050, 2100).
RF-reverse: the RF estimates based on NCEP diffuse and direct radiation ratios for the present (2018) and reversed NCEP diffuse/direct ratios for the future (2050, 2100).
RF-CMIP5: the RR estimates based on NCEP diffuse and direct radiation ratios for the current (2018) and projected diffuse/direct ratios for the future (2050, 2100) provided by three models ensemble means (CANESM2, NOESM_M, and NORESM_ME).

Supplementary Table 4 Overview of GCMs considered in this paper for snow cover and solar radiation projections.

Institution	Model ID	References
Canadian Centre for Climate Modelling and Analysis	CanESM2	Hua, et al.[6]
Centre National de Recherches Meteorologiques	CNRM-CM5	Voldoire, et al.[7]
Institute of Atmospheric Physics	FGOALS-g2	Li, et al.[8]
Goddard Institute for Space Studies	GISS-E2-H	Schmidt, et al.[9]
Goddard Institute for Space Studies	GISS-E2-R	Schmidt, et al.[9]
Atmosphere and Ocean Research Institute	MIROC5	Watanabe, et al.[10]
Japan Agency for Marine-Earth Science and Technology	MIROC-ESM	Watanabe, et al.[11]
Japan Agency for Marine-Earth Science and Technology	MIROC-ESM-CHEM	Watanabe, et al.[11]
Max Planck Institute for Meteorology	MPI-ESM-LR	Raddatz et al.[12]
Max Planck Institute for Meteorology	MPI-ESM-MR	Raddatz et al.[13]
Meteorological Research Institute	MRI-CGCM3	Yukimoto, et al.[13]
Norwegian Climate Centre	NorESM1-M	Bentsen, et al.[14]
Norwegian Climate Centre	NorESM1-ME	Bentsen, et al.[14]

Supplementary Table 5. log normal distributions used to approximate uncertainties around radiative forcing (RF) and equilibrium climate sensitivity (ECS). RF is scaled up by 1000 in lognormal distributions.

Quantities	Mean and 90% interval	Approximate log normal distribution
ECS	3 [2, 5]	Lognormal (1.057983, 0.2850598)
RF in 2050 relative to 2018 under SSP1-2.6	0.00128 [0.00102,0.00206]	Lognormal (0.2183069, 0.2389693)
RF in 2050 relative to 2018 under SSP2-4.5	0.00133 [0.00107, 0.00202]	Lognormal (0.2652347, 0.199721)
RF in 2050 relative to 2018 under SSP5-8.5	0.00158 [0.00123, 0.00240]	Lognormal (0.4359616, 0.2071869)
RF in 2100 relative to 2018 under SSP1-2.6	0.00157 [0.00119,0.00255]	Lognormal (0.4168647,0.2615758)
RF in 2100relative to 2018 under SSP2-4.5	0.00187 [0.00144, 0.00288]	Lognormal (0.5995654, 0.2296652)
RF in 2100 relative to 2018 under SSP5-8.5	0.00307 [0.00233, 0.00452]	Lognormal (1.098737, 0.214197)

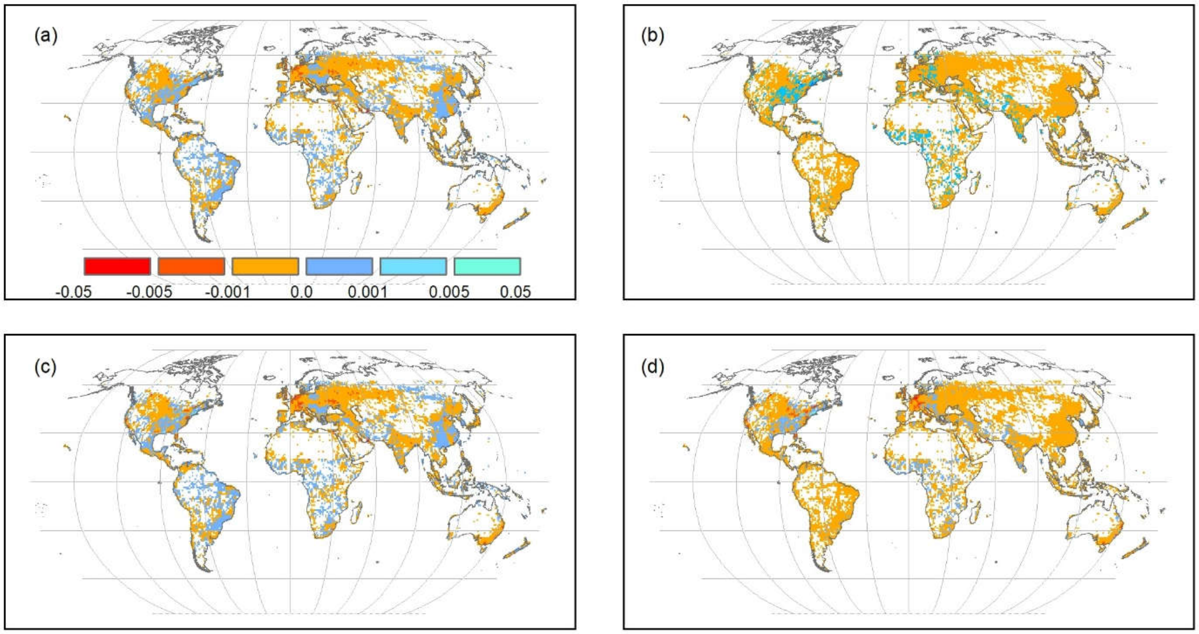

Supplementary Figure 1 Global change in land surface albedo due to urbanization. Global change in land surface albedo due to urbanization in 2050 relative to 2018 (a) and in 2100 relative to 2050 (b) under SSP1 scenario, and in 2050 relative to 2018 (c) and in 2100 relative to 2050 (d) under SSP5-8.5 scenario.

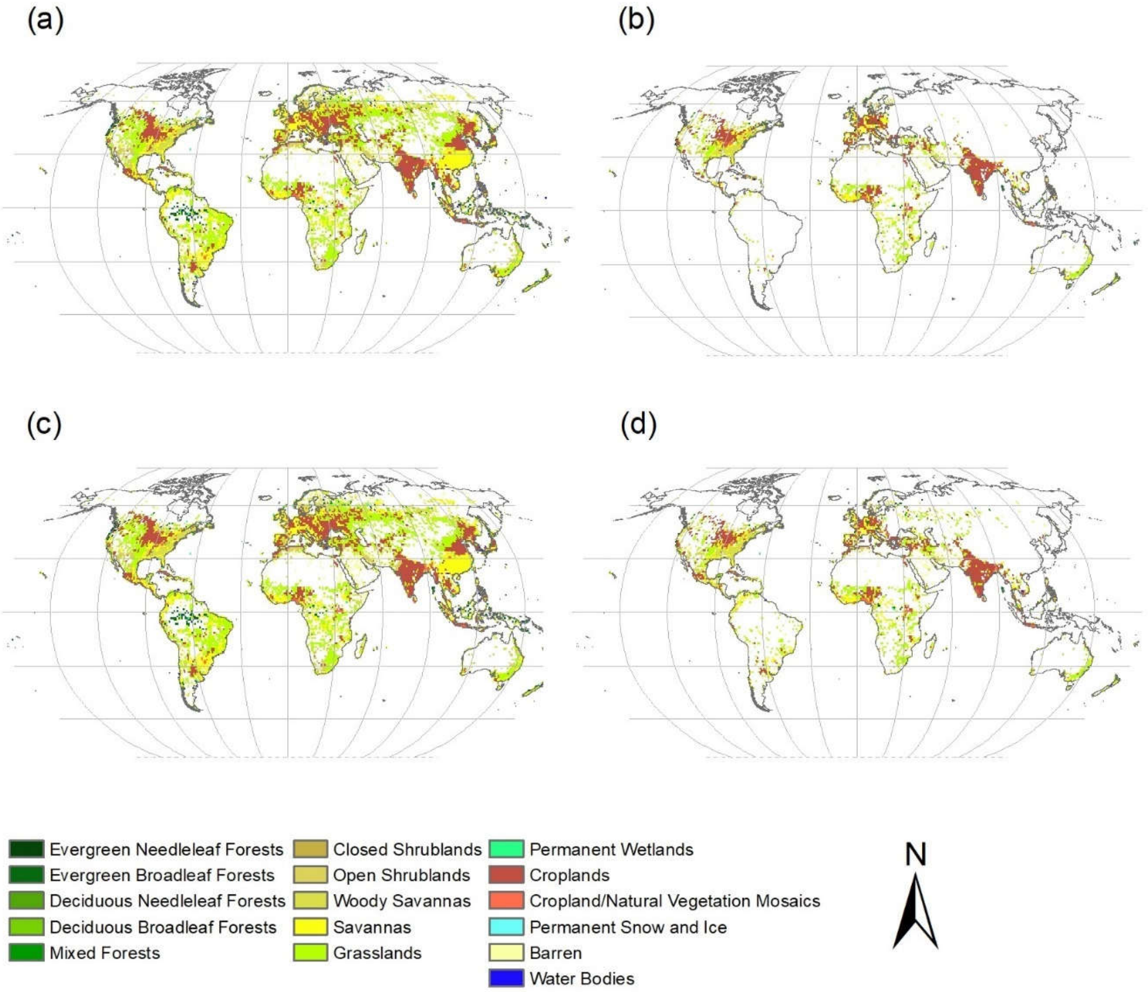

Supplementary Figure 2 Dominant land cover converted to urban land. The land cover types with the most area being converted into urban land at grid level in 2050 relative to 2018 (a) and in 2100 relative to 2018 (b) under SSP1 urbanization scenario, and in 2050 relative to 2018 (c) and in 2100 relative to 2018 (d) under SSP5 urbanization scenario.

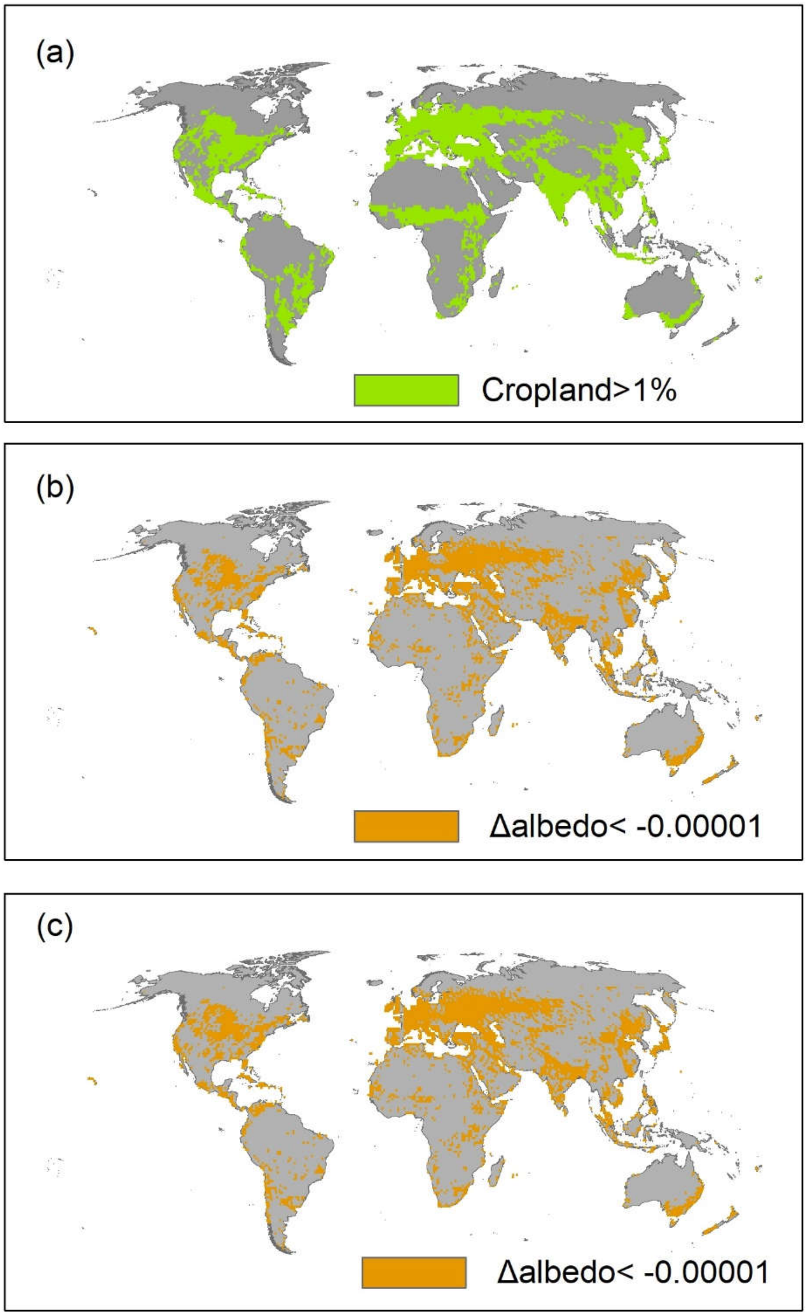

Supplementary Figure 3 Comparison of spatial patterns of cropland and albedo changes. Comparison of the spatial pattern of (a) global cropland (areal percentage >1%) in 2010 based on MODIS land use land cover product[5] and (b) change of albedo in 2050 relative to 2018 and (c) change of albedo in 2100 relative to 2018 caused by projected urban expansion under SSP2-2.6 scenario.

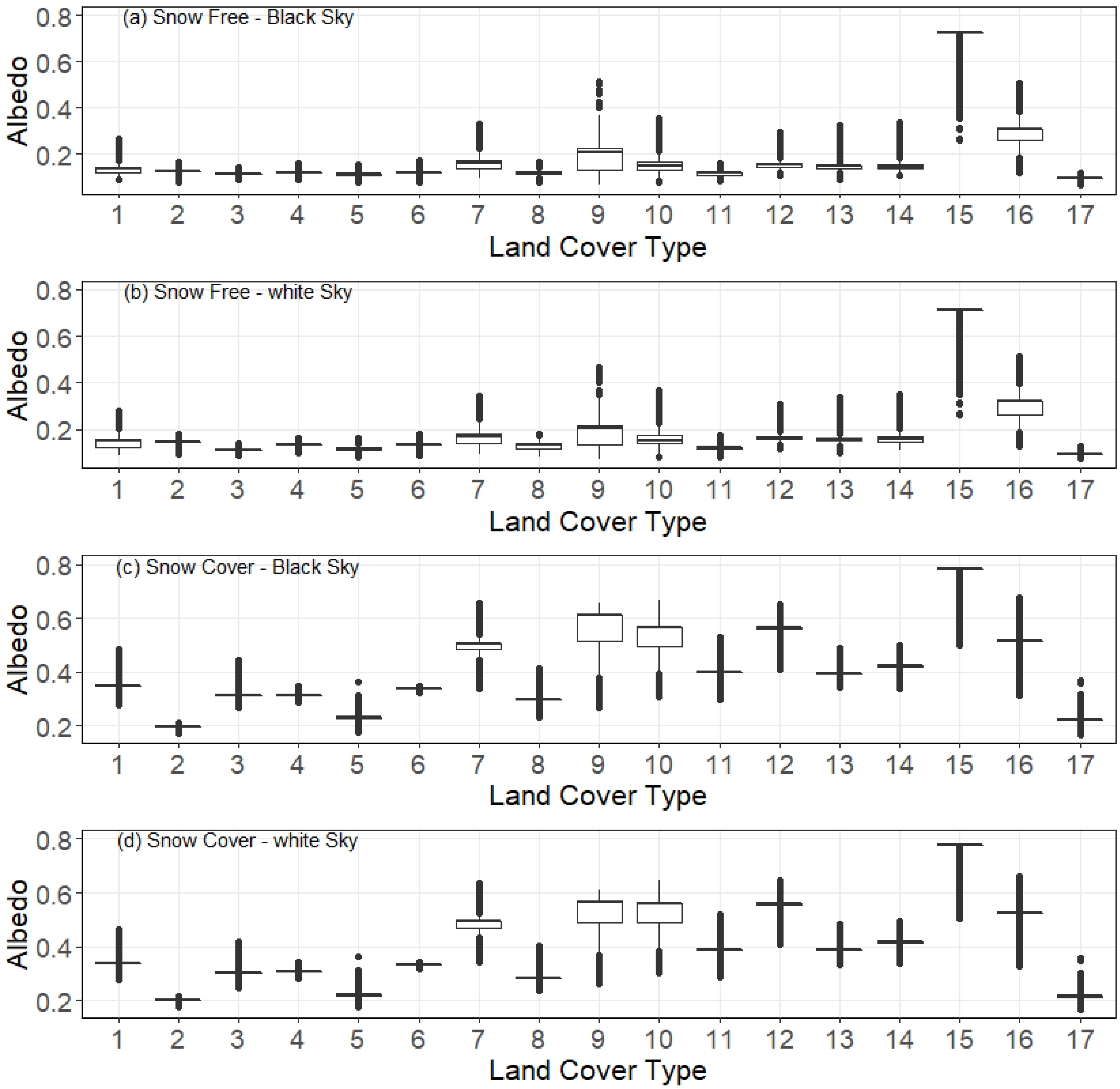

Supplementary Figure 4 Comparison of the annual mean albedo among different land cover types. The boxplots are drawn based on 17726 data points extracted from all 1°×1° land pixels. See Table S1 for the description of each numbered land cover. (a) Black-sky albedo under snow free conditions, (b) White-sky albedo under snow free conditions, (c) Black-sky albedo under snow cover conditions, and (d) White-sky albedo under snow cover conditions.

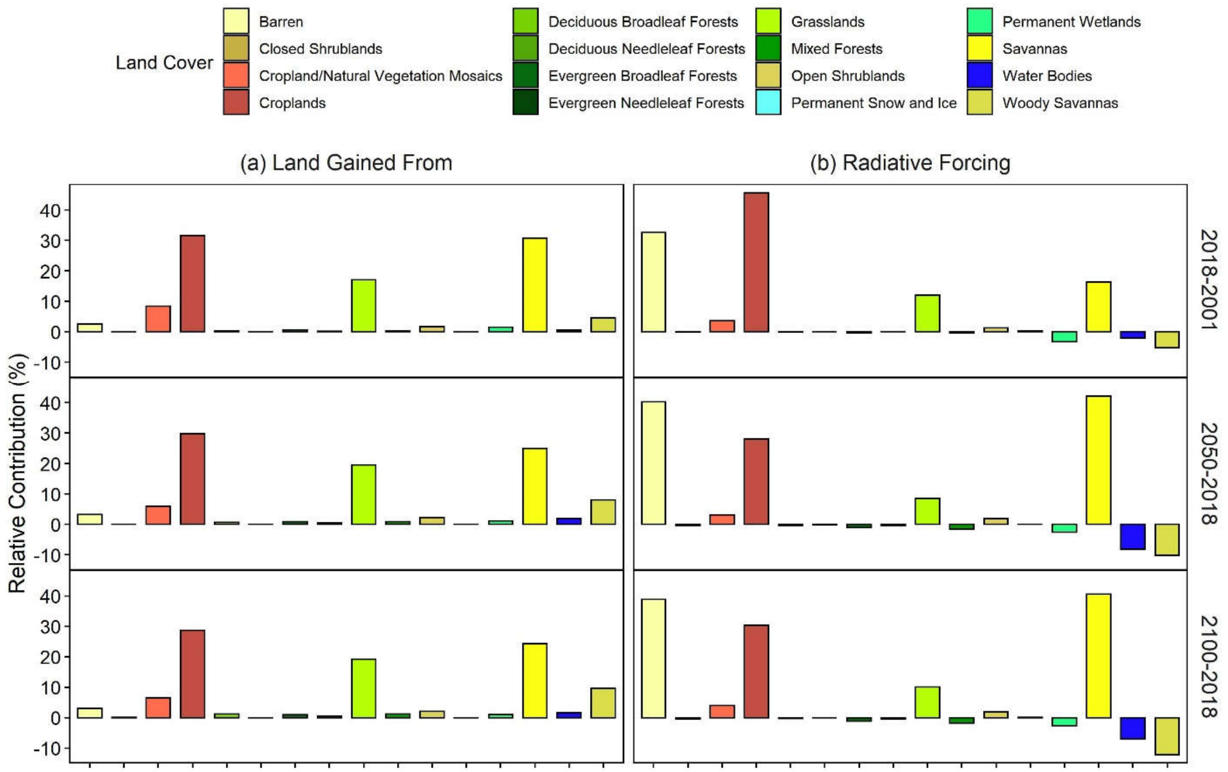

Supplementary Figure 5 Contributions of different land cover types to gained urban land and radiative forcing under SSP1-2.6. Contributions of different land cover types to (a) the global total albedo-induced warming potential and (b) the global total new urban land areas in 2018 relative to 2001(2018–2001), in 2050 relative to 2018 (2050–2018), and in 2100 relative to 2018 (2100–2018). Data for future periods are based on SSP1-2.6 scenario.

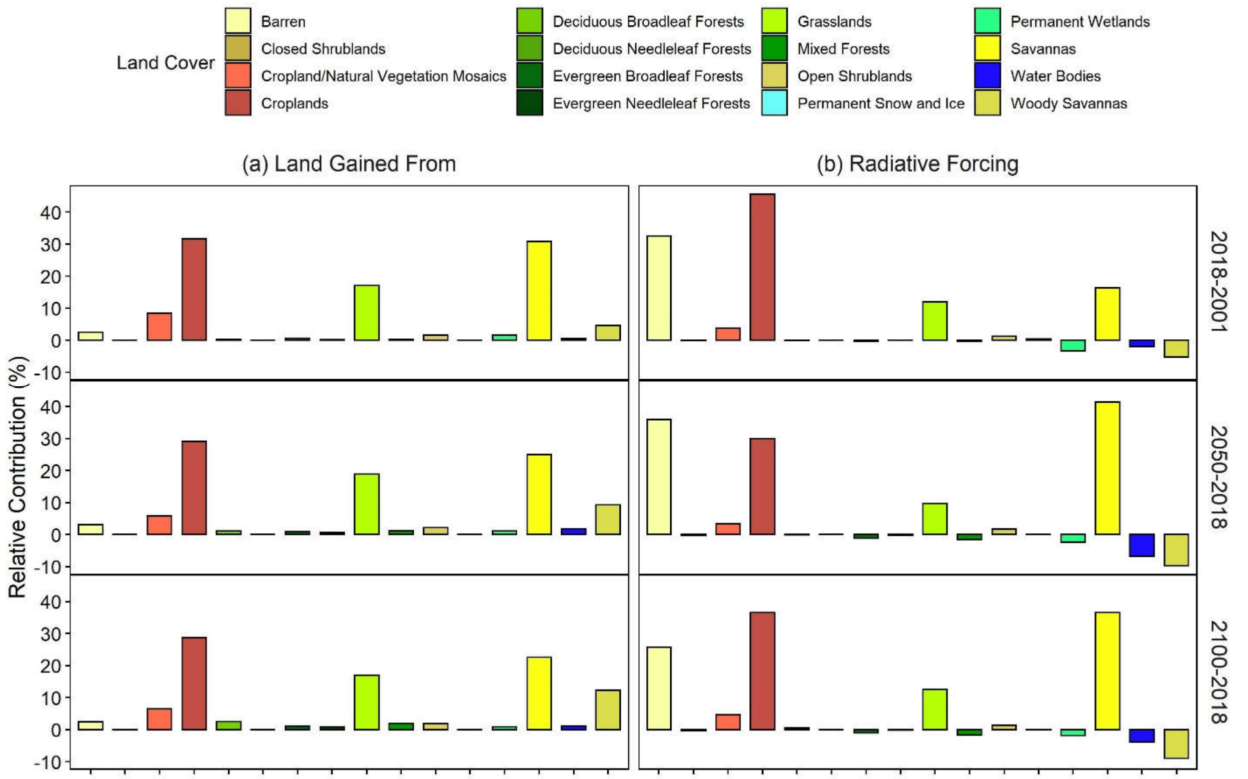

Supplementary Figure 6 Contributions of different land cover types to gained urban land and radiative forcing under SSP5-8.5. Contributions of different land cover types to (a) the global total albedo-induced warming potential and (b) the global total new urban land areas in 2018 relative to 2001(2018–2001), in 2050 relative to 2018 (2050–2018), and in 2100 relative to 2018 (2100–2018). Data for future periods are based on SSP5-8.5 scenario.

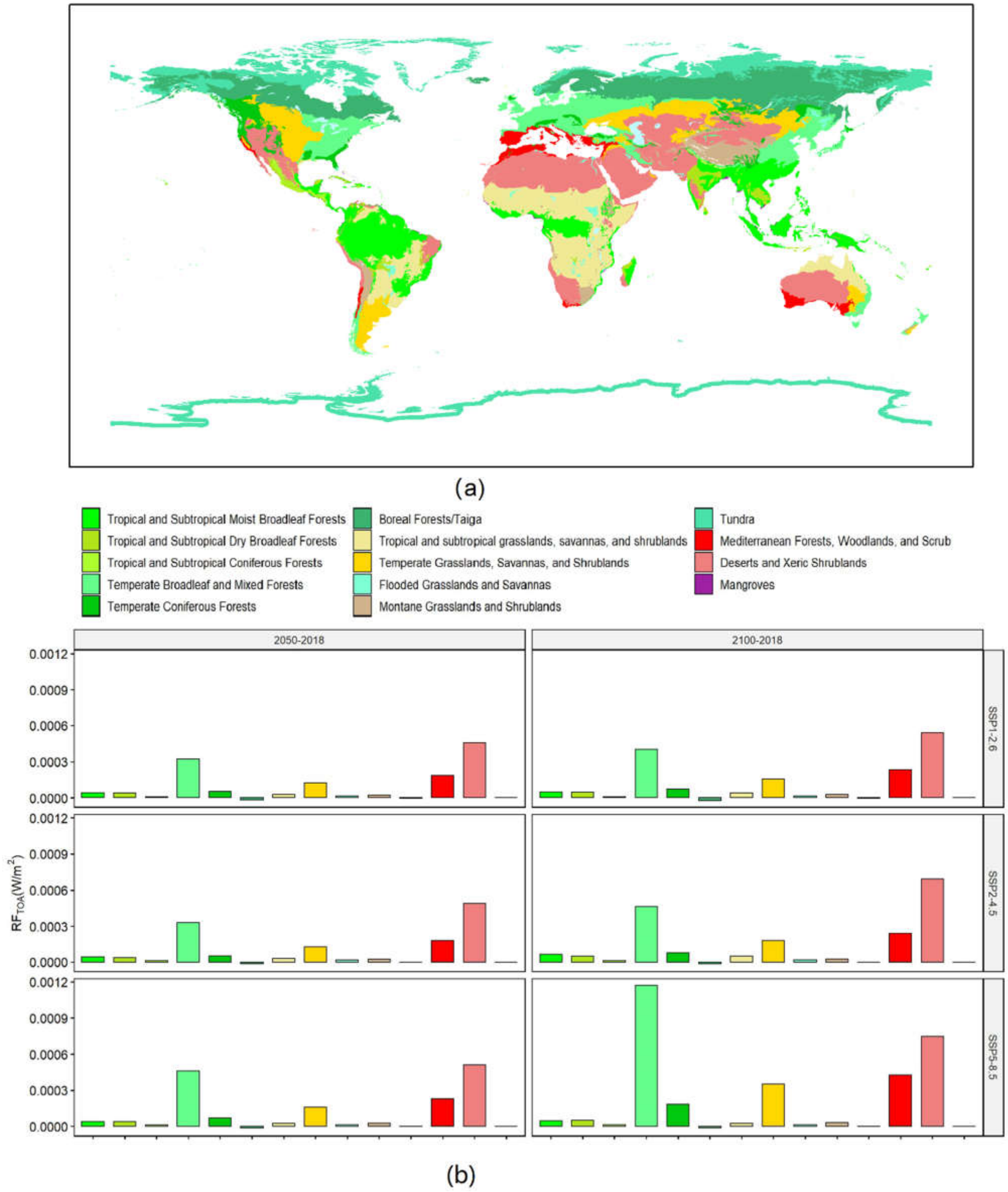

Supplementary Figure 7 Global total albedo-induced warming potential in different biome. (a) Global division of biome[15] and (b) global total albedo-induced warming potential (top-of-atmosphere radiative forcing) in different biomes due to urbanization in 2050 relative to 2018 (2050–2018), and in 2100 relative to 2018 (2100–2018) under three illustrative scenarios (SSP1-2.6, SSP2-4.5, and SSP5-8.5).

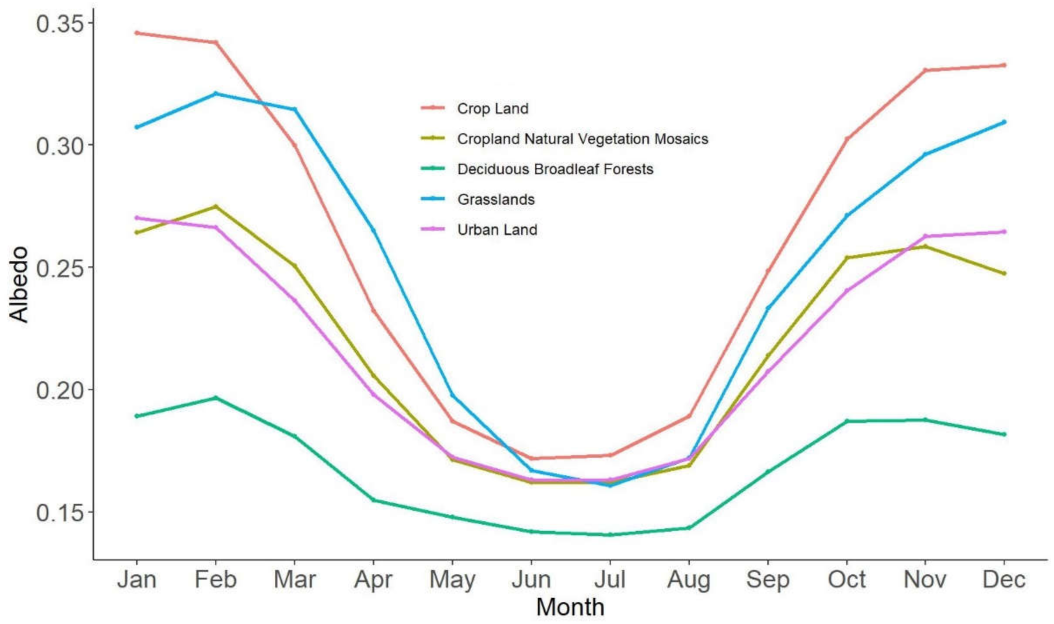

Supplementary Figure 8 Seasonal change of albedo. The seasonal change of global average albedo of cropland, cropland natural vegetation mosaics, deciduous broadleaf forests, grassland, and urban land. Data are derived based on long-term (2001-2010) averages of MODIS observations.

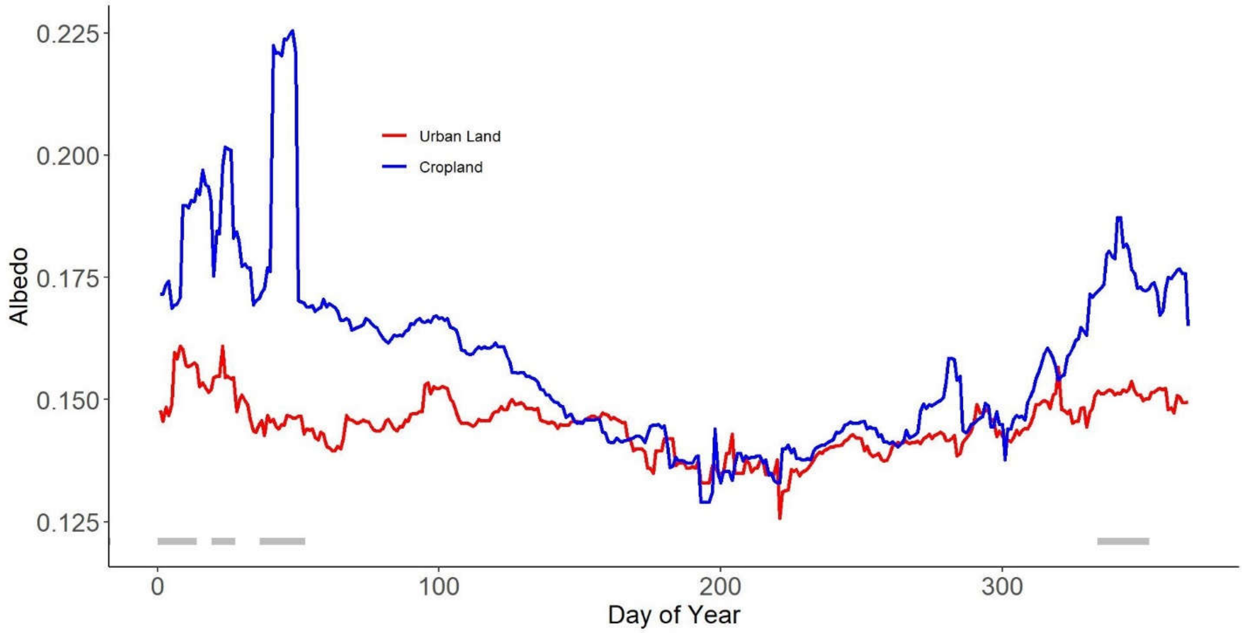

Supplementary Figure 9. Seasonal change of albedo and snow effect. The seasonal change of MODIS pixel-wise shortwave albedo at cropland (39.679,116.221) and urban land (40.086,116.452) at a local location in Beijing Metropolitan area. Data are derived based on long-term (2015-2020) averages of MODIS observations. The grey horizontal bars depict snowing periods.

Supplementary References:

1. Guo, T. et al. Multi-decadal analysis of high-resolution albedo changes induced by urbanization over contrasted Chinese cities based on Landsat data. Remote Sensing of Environment. 269, 112832 (2022).
2. Hartmann, D. L. (2016). *Global Physical Climatology*. 2nd edition, Amsterdam, Netherlands, Elsevier.
3. Cronin, T. W. (2014). On the choice of average solar zenith angle. *Journal of the Atmospheric Sciences*, 71(8), 2994–3003.
4. Riahi, Keywan, et al., (2017). The Shared Socioeconomic Pathways and their energy, land use, and greenhouse gas emissions implications: An overview, Global Environmental Change, 42, 153-168
5. Sulla-Menashe and Friedl, (2018). User Guide to Collection 6 MODIS Land Cover (MCD12Q1 and MCD12C1) Product https://lpdaac.usgs.gov/sites/default/files/public/product_documentation/mcd12_user_guide_v6.pdf (2018)
6. Hua, Wenjian., et al., (2015). Assessing climatic impacts of future land use and land cover change projected with the CanESM2 model. International Journal of Climatology, 35(12), 3661-3675.
7. Voldoire, Aurore, et al., (2013). The CNRM-CM5. 1 global climate model: description and basic evaluation. Climate Dynamics, 40 (9), 2091-2121.
8. Li, Lijuan, et al., (2013). The flexible global ocean-atmosphere-land system model, grid-point version 2: FGOALS-g2. Advances in Atmospheric Sciences, 30 (3), 543-560.
9. Schmidt, Gavin A., et al., (2014). Configuration and assessment of the GISS ModelE2 contributions to the CMIP5 archive. Journal of Advances in Modeling Earth Systems, 6, 141-184.
10. Watanabe, Masahiro et al., (2010). Improved climate simulation by MIROC5: Mean states, variability, and climate sensitivity. Journal of Climate, 23, 6312-6335
11. Watanabe, S., et al., (2011). MIROC-ESM: model description and basic results of CMIP5-20c3m experiments, Geoscientific Model Development., 4, 1063-1128
12. Raddatz et al., (2007). Will the tropical land biosphere dominate the climate-carbon cycle feedback during the twenty first century? Climate Dynamics, 29, 565-574
13. Yukimoto, Seiji, et al., (2012). A new global climate model of the Meteorological Research Institute: MRI-CGCM3—Model description and basic performance. Journal of the Meteorological Society of Japan. 90, 23-64.
14. Bentsen, Mats, et al., (2013). The Norwegian earth system model, NorESM1-M—Part 1: Description and basic evaluation of the physical climate Geoscientific Model Development 6(3), 687-720.
15. Olson, D. M., et al., (2001). Terrestrial ecoregions of the world: a new map of life on Earth. Bioscience 51(11):933-938
